# Pulmonary function in school-age children following intravitreal injection of bevacizumab for retinopathy of prematurity

**DOI:** 10.1038/s41598-022-22338-2

**Published:** 2022-11-05

**Authors:** Ching-Yen Huang, Shen-Hao Lai, Hsiao-Jung Tseng, Tsung-Chieh Yao, Wei-Chi Wu

**Affiliations:** 1 Department of Ophthalmology, Chang Gung Memorial Hospital, 5 Fu-Hsin Street, Kweishan, Taoyuan, 33305 Taiwan; 2 College of Medicine, Chang Gung University, Taoyuan, Taiwan; 3 Division of Pulmonology, Department of Pediatrics, Chang Gung Memorial Hospital, Taoyuan, Taiwan; 4 Clinical Trial Center, Biostatistics Unit, Chang Gung Memorial Hospital, Taoyuan, Taiwan; 5 Division of Allergy, Asthma, and Rheumatology, Department of Pediatrics, Chang Gung Memorial Hospital, 5 Fu-Hsin Street, Gueishan, Taoyuan, 33305 Taiwan

**Keywords:** Retinopathy of prematurity, Respiratory signs and symptoms

## Abstract

The effect of anti-vascular endothelial growth factor on neonatal lung development was inconclusive. To evaluate pulmonary function in school-age children who have received intravitreal bevacizumab (IVB) for retinopathy of prematurity (ROP), this study included 118 school-aged children who were grouped into three groups: full-term control children (group 1), preterm children who had not received IVB treatment (group 2) and preterm children with ROP who had received IVB treatment (group 3). Pulmonary function was measured by spirometry and impulse oscillometry. Pulmonary function was significantly better in group 1 than in groups 2 and 3 (all p < 0.05 in forced vital capacity (FVC), forced expiratory volume in 1 s (FEV_1_), forced expiratory flow between 25 and 75% of FVC (FEF_25–75_), and respiratory resistance at 5 Hz and difference between respiratory resistance at 5 and 20 Hz (R5-R20). There were no statistically significant differences between group 2 and group 3 in all pulmonary function parameters, including FVC, FEV_1_, ratio of FEV_1_ to FVC, FEF_25-75_, R5, R20, R5–R20, and respiratory reactance at 5 Hz. In conclusion, our study revealed that preterm infants receiving IVB for ROP had comparable pulmonary function at school age to their preterm peers who had not received IVB treatment.

## Introduction

Retinopathy of prematurity (ROP) is an important infantile retinal disease that causes blindness or severe visual impairment in childhood^1,2^ and imposes a heavy financial burden in several countries^3^. Intravitreal anti-vascular endothelial growth factor (anti-VEGF) with bevacizumab monotherapy, compared with conventional laser therapy in infants with stage 3 + retinopathy of prematurity, showed a significant benefit for zone I disease^4^. Compared with traditional laser monotherapy, intravitreal injection of anti-VEGF (IVI) reduced the recurrence of zone I ROP and the risk of high myopia^5–7^. When combined with laser therapy, anti-VEGF reduced the risk of retinal detachment^6^. Several studies also proposed that patients who had received anti-VEGF treatment had favorable anatomical outcomes^8–10^.

Regarding the safety issues of using anti-VEGF for ROP, potential adverse events include endophthalmitis, intraocular inflammation and rhegmatogenous retinal detachment^11,12^. Regarding the systemic safety of this treatment, VEGF plays a crucial role in the development of several organs, including the lungs, brain, kidneys, and liver^13^. Multiple studies have evaluated the effect of anti-VEGF treatment on the brain, that is, neurodevelopmental outcomes^14–20^. While some studies presented negative results^19,20^, most studies revealed difference in neurodevelopment after anti-VEGF treatment^14–18^. Among all organs, the lungs contain the highest level of VEGF transcripts^12^. An animal study also revealed that the blockage of VEGF impaired lung development^13^. Since serum VEGF levels are reduced for up to 8 weeks after intravitreal anti-VEGF treatment for ROP^21–23^, additional impacts on lung function are a concern in neonates with a history of anti-VEGF treatment. The hypothesis of our study was that the reduction in serum VEGF levels may have an impact on lung development. To date, no clinical study has assessed the long-term pulmonary function of premature patients after they have received anti-VEGF treatment for ROP. Therefore, this study aimed to investigate the pulmonary function of school-age children with or without a history of anti-VEGF treatment for ROP during infancy.

## Methods

### Study subjects and enrollment and exclusion criteria

This study was approved by the institutional review board of Chang Gung Memorial Hospital in Taoyuan, Taiwan (contract, IRB201900571B0) and adhered to the tenets of the Declaration of Helsinki. Written informed consent was obtained from each patient’s parent for the enrollment of his or her child in the study.

**Figure 1:**
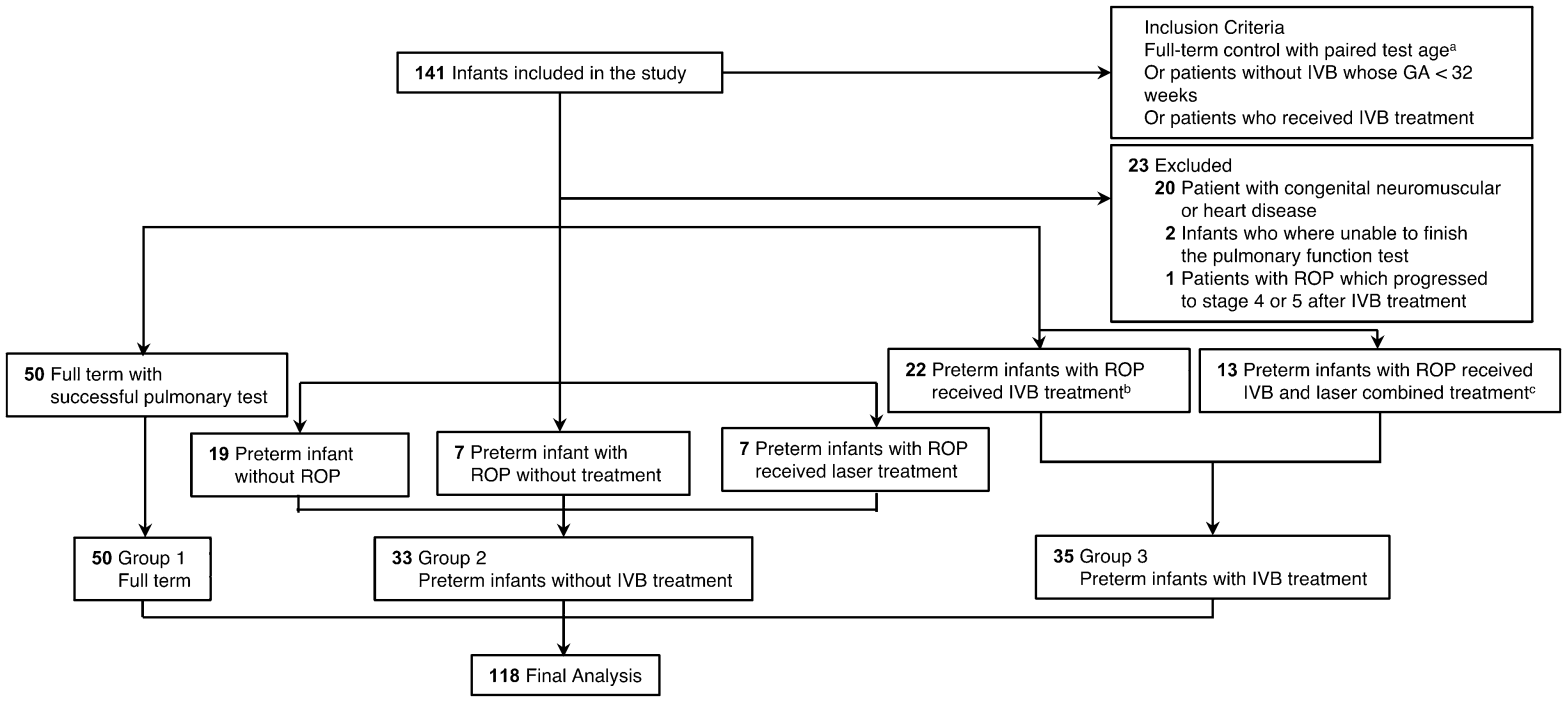
Flowchart showing the inclusion and exclusion of patients during the study period. Abbreviations: IVB, intravitreal injection of bevacizumab; ROP, retinopathy of prematurity. ^a^Full-term children were matched to groups 2 and 3 by age at pulmonary function testing. ^b^One patient received IVB in the right eye and laser treatment in the left eye. ^c^One patient received IVB in the right eye and combined IVB and laser treatment in the left eye.

This retrospective case–control study was conducted between 2016/07/01 and 2020/07/31 at Chang Gung Memorial Hospital, Taoyuan, Taiwan. Three groups of subjects were enrolled in this study: Group 1 consisted of full-term children without ROP, matched to groups 2 and 3 by age at pulmonary function testing, who underwent baseline pulmonary function testing in the Longitudinal Investigation of Global Health in Taiwanese Schoolchildren (LIGHTS) cohort^24–28^. Group 2 consisted of preterm children with no history of intravitreal bevacizumab (IVB) treatment, and this group was composed of three parts: patients without ROP, patients with untreated ROP, and patients who received laser treatment. Group 3 consisted of premature children with ROP who received anti-VEGF treatment, composed of IVB monotherapy or combined IVB and laser therapy (Fig. 1). The indication for treatment was type 1 ROP as defined by the Early Treatment for ROP Study^29,30^, that is, zone I ROP of any stage with plus disease (a degree of dilation and tortuosity of the posterior retinal blood vessels meeting or exceeding that of a standard photograph), zone I stage 3 ROP without plus disease, or zone II stage 2 or 3 ROP with plus disease^29,30^. The technique used for IVB was described previously^31,32^; that is, 0.625 mg (0.025 mL) bevacizumab was injected intravitreally via the pars plicata under intravenous sedation. For IVB or laser treatment decisions, we suggested that patients with zone I ROP receive IVB treatment according to the BEAT ROP study results^4^. For type 1 ROP patients with postmenstrual age over 40 weeks, we suggested laser therapy because IVB therapy may increase the risk of tractional retinal detachment^33^. However, the final decision of IVB or laser treatment was made through shared decision-making after fully explaining the benefits and risks to the patients’ families.

Full-term birth was defined as birth later than 37 weeks of gestational age (GA). Parents of premature children who had been admitted to neonatal intensive care units before and were attending follow-up outpatient clinics were invited to join this study. The status of the off-label use of IVB for ROP treatment was explained to the parents in detail. Pulmonary function testing was arranged for the school-age patients. Preterm patients with prior ROP and IVB treatment were allocated to the target study group (Group 3). Preterm patients without IVB who had been born before 32 weeks of GA (Group 2) were selected for similarity to Group 3 in GA. Full-term children (Group 1) matched to groups 2 and 3 by age at pulmonary testing were enrolled because pulmonary function was highly correlated with age^34–36^. Patients who were unable to finish the pulmonary function test, full-term patients with congenital cardiac or neuromuscular disease and patients whose ROP progressed to stage 4 or 5 after IVB or laser treatment were excluded (Fig. 1).

### Pulmonary function measurement

Spirometry is the gold standard and the most commonly used, well-established tool to detect lung disease^36^. In our study, forced vital capacity (FVC), forced expiratory volume in 1 s (FEV_1_), ratio of FEV_1_ to FVC (FEV_1_/FVC), and forced expiratory flow between 25 and 75% (FEF_25–75_) were measured by using a spirometer (Spirolab II, Medical International Research, Rome, Italy). The measurements of spirometry were described in a prior publication^34,37^.

An impulse oscillometer (IOS) is a noninvasive tool that is increasingly used in children to measure respiratory resistance (Rrs) and reactance (Xrs)^38^. We used a commercially equipped IOS (MasterScreen; Jaeger, Wurzburg, Germany). We used oscillometry procedures that have previously been described in detail^35^. The Rrs was recorded at 5 Hz (R5: representing total airway resistance) and 20 Hz (R20: representing proximal airway resistance). The difference between R5 and R20 was calculated (R5–R20: representing peripheral airway resistance). The Xrs was recorded at 5 Hz (X5).

### Statistical analysis

Group differences in continuous variables were identified by using one-way ANOVA. Group differences in categorical variables were identified by using the chi-squared test or Fisher’s exact test when applicable. Univariable linear regression analyses were performed to determine the relationships between individual variables and pulmonary function parameters. Factors that were significant in the univariate analysis were used for a multivariable pretest, and only factors that were still significant were used in the final multivariable analysis. Final multivariable regression analyses were performed to estimate the relationships between variables of interest and pulmonary function and the potential interaction between significant variables in the univariable linear regression. The significance threshold for all tests was set at *P* < 0.05. All statistical analyses were performed with SPSS software (IBM SPSS Statistics for Windows, Version 22.0. Armonk, NY: IBM Corp.).

## Results

### Study participants

One hundred eighteen children (65 boys and 53 girls; mean age, 6.67 ± 0.52 years) were included in the final analysis. A flowchart showing how patients were enrolled and excluded from the study is presented in Fig. 1. Among these children’s perinatal history, 50 were full-term infants matched with the preterm infants by test age (Group 1), 33 were preterm infants with no history of IVB treatment (Group 2), and 35 were preterm infants with a history of IVB treatment (Group 3). The ages of the children at testing were similar among 3 groups (*p* = 0.58). Table 1 shows the demographic data and important confounding factors related to pulmonary function and test status. Both group 2 and group 3 showed less favorable baseline birth demographics and more perinatal complications than group 1. Group 3 showed lower GA (*P* < 0.001), lower birth weight (BW) (*P* < 0.001), a lower rate of cesarean section (CS) (*P* = 0.04), a lower rate of premature rupture of membrane (PROM) (*P* = 0.03), a lower rate of paternal asthma (P = 0.02), a higher rate of surfactant use (*P* = 0.005), a higher rate of bronchopulmonary dysplasia (BPD) (*P* = 0.03), and a longer duration of mechanical ventilator use after birth (*P* < 0.001) than group 2.

**Table 1:** Demographics of study participants.

	Full-term control (Group 1; *n* = 50)	Prematurity without IVB treatment (Group 2; *n* = 33)	Prematurity with IVB treatment (Group 3; *n* = 35)	*P* value (*F*-value)	Group 2 versus. 3
Age at pulmonary function test, mean ± SD (min, max)	6.62 ± 0.22 (6.8, 7.9)	6.69 ± 0.59 (5.7, 7.8)	6.73 ± 0.64 (6.3, 7.4)	0.58^a^ (0.6)	1.00
Height at test (cm), mean ± SD (min, max)	119.9 ± 4.5^c^ (106, 131)	115.7 ± 6.3^c^ (105, 128)	116.8 ± 7.0 (103, 133)	0.004^a^ (3)	1.00
Body weight at test (kg), mean ± SD (min, max)	22.4 ± 4.1 (17, 37)	21.1 ± 4.7 (15, 35)	20.7 ± 3.9 (14, 34)	0.16^a^(1)	1.00
BMI at test, mean ± SD (min, max)	15.5 ± 2.4 (12, 24)	15.6 ± 2.3 (12, 23)	15.1 ± 2.0 (13, 22)	0.56^a^ (1)	0.99
Sex, male (%)	26 (52.0)	16 (48.5)	23 (65.7)	0.31^b^	0.20
Gestational age (weeks), mean ± SD (min, max)	38.8 ± 1.0^c,d^ (37, 42)	28.7 ± 2.4^c,e^ (24, 32)	25.8 ± 1.4^d,e^ (23, 30)	< 0.001^a^ (1409)	< 0.001^e^
Birth weight (g), mean ± SD (min, max)	3070.7 ± 409.5^c,d^ (2075, 4010)	1180.9 ± 453.4^c,e^ (582, 2120)	795.1 ± 156.8^d,e^ (505, 1338)	< 0.001^a^ (752)	< 0.001^e^
Cesarean section, no. (%)	17 (34.0)^c,d^	24 (85.7)^c,e^	23 (65.7)^d,e^	< 0.001^b^	0.04^e^
PROM, no. (%)	0 (0.0)^c,d^	16 (48.5)^c,e^	9 (25.7)^d,e^	< 0.001^b^	0.03^e^
Received antenatal steroids	0 (0.0)^c,d^	18 (54.5)^c^	20 (57.1)^d^	< 0.001^b^	0.99
Received surfactant	0 (0.0)^c,d^	15 (45.5)^c,e^	27 (77.1)^d,e^	< 0.001^b^	0.005^e^
PHTN, no. (%)	0 (0.0)	0 (0.0)	2 (5.7)	0.15^b^	0.17
BPD, no (%)	0 (0.0)^c,d^	13 (39.4)^c,e^	23 (65.7)^d,e^	< 0.001^b^	0.03^e^
BPD, mild	0 (0.0)	4 (12.1)	1 (2.9)		
BPD, moderate	0 (0.0)	3 (9.1)	12 (34.3)		
BPD, severe	0 (0.0)	6 (18.2)	10 (28.6)		
Sepsis, no. (%)	0 (0.0)^c,d^	8 (24.2)^c^	12 (34.3)^d^	< 0.001^b^	0.41
Hospital duration (days)^f^, mean ± SD	4.0 ± 1.7	73.5 ± 39.2	121.1 ± 29.7	< 0.001^a^ (437)	< 0.001^e^
Mechanical ventilator duration (days)^g^, mean ± SD	0.1 ± 0.4^c,d^	32.3 ± 35.1^c,e^	72.9 ± 18.9^d,e^	< 0.001^a^ (249)	< 0.001^e^
Hospitalization due to pulmonary infection^h^, no. (%)	5 (10.0)^c,d^	12 (36.4)^c^	14 (40.0)^d^	0.003^b^	0.83
Asthma	6 (12.2)	6 (18.2)	2 (5.7)	0.29^b^	0.10
Paternal asthma, no. (%)	3 (6.0)	5 (15.2)^e^	0 (0.0)^e^	0.04^b^	0.02^e^
Maternal asthma, no. (%)	0 (0.0)^d^	2 (6.1)	5 (14.3)^d^	0.01^b^	0.28
Maternal smoking at pregnancy, no (%)	0 (0.0)	0 (0.0)	1 (2.9)	0.57^b^	0.34

*BMI* body mass index; *BPD* bronchopulmonary dysplasia; *IVB* intravitreal injection of Bevacizumab; *PHTN* pulmonary hypertension; *PROM* premature rupture of membrane; *RDS* respiratory distress syndrome; *SD* standard deviation.

^a^*P* values calculated by analysis of variance and post hoc tests performed by the Bonferroni test. ^b^*P* values calculated by chi-square test or Fisher exact test. ^c^Significant difference between groups 1 and 2. ^d^Significant difference between groups 1 and 3. ^e^Significant difference between groups 2 and 3. ^f^Duration of total hospital stay from birth to discharge (3 patients was lack of data and excluded from analysis due to birth at other medical cencter). ^g^Duration of received mechanical ventilator after birth. ^h^Hospitalization due to pulmonary infection within three years old.

Supplementary table S1 shows the remaining demographics, confounding factors, ROP disease status and treatment status. Group 3 showed lower Apgar scores (*P* = 0.001 and *P* = 0.02 at 1 min and 5 min, respectively), a higher incidence of respiratory distress syndrome (RDS) (*P* = 0.007), a lower incidence of atopic dermatitis (*P* = 0.003), a higher rate of paternal smoking during pregnancy (*P* = 0.007), and a longer breastfeeding duration (*P* = 0.03) than group 2. There were also significant differences between groups 2 and 3 in terms of ROP stage (*P* < 0.001), zone (*P* < 0.001), plus disease (*P* < 0.001) and treatment received (*P* < 0.001).

### Pulmonary function outcomes

Table 2 summarizes the pulmonary function outcomes of the three groups, including FVC, FEV_1_, FEV_1_/FVC, FEF_25-75_, R5, R20, X5 and R5-R20. There were no significant differences among the 3 groups in FEV_1_/FVC, R20 or X5. However, FVC, FEV_1_, FEF_25–75_, R5, and R5–R20 showed significant differences. Compared with group 1, group 2 and group 3 both showed significantly reduced FVC, reduced FEV_1_, increased R5 and increased R5–R20. Additionally, group 3 showed lower FEF_25–75_ than group 1. Compared with group 2, group 3 showed trends toward reduced FVC, FEV_1_, FEV_1_/FVC, and FEF_25–75_ and increased R5, R20, and R5-R20. However, the post hoc tests showed that there were no statistically significant differences in any pulmonary outcome parameters between group 2 and group 3.

**Table 2:** Pulmonary function outcomes.

	Full-term control (Group 1; *n* = 50)	Prematurity without IVB treatment (Group 2; *n* = 33)	Prematurity with IVB treatment (Group 3; *n* = 35)	*p* value (*F* value)	Post-hoc (Group 2 vs. 3)
**Spirometry**					
FVC (L), mean ± SD	1.26 ± 0.27^a,b^	1.06 ± 0.25^a^	0.98 ± 0.26^b^	< 0.001 (13)	0.63
FEV_1_ (L), mean ± SD	1.15 ± 0.26^a,b^	0.98 ± 0.23^a^	0.89 ± 0.24^b^	< 0.001 (13)	0.37
FEV_1_/FVC (%), mean ± SD	91.42 ± 6.61	92.38 ± 6.00	90.60 ± 8.40	0.57 (0.5)	0.88
FEF_25-75_, mean ± SD	1.57 ± 0.52^b^	1.35 ± 0.40	1.12 ± 0.36^b^	< 0.001 (10)	0.11
**Impulse oscillometry**					
R5 kPa/(L/s)	9.52 ± 2.28^a,b^	10.98 ± 2.44^a^	11.09 ± 2.60^b^	0.005 (6)	1.00
R20 kPa/(L/s)	6.56 ± 1.40	6.90 ± 1.82	6.98 ± 1.44	0.21 (2)	1.00
X5 kPa/(L/s)	− 2.84 ± 1.34	− 3.07 ± 1.75	− 2.59 ± 2.00	0.53 (0.6)	0.79
R5-R20 kPa/(L/s)	2.96 ± 1.63^a,b^	4.08 ± 1.48^a^	4.12 ± 1.94^b^	0.002 (7)	1.00

*FEF*_25–75_ forced expiratory flow between 25 and 75% of FVC; *FEV*_1_ forced expiratory volume in 1 second; *FEV*_1_*/FVC* ratio of FEV_1_ to FVC; *FVC* forced vital capacity; *IVB* intravitreal injection of Bevacizumab; *R5* respiratory resistance at 5 Hz; *R20* respiratory resistance at 20 Hz; *R5–R20* difference between respiratory resistance at 5 Hz and 20 Hz; *SD* standard deviation; *X5* respiratory reactance at 5 Hz.

*P* values calculated by analysis of variance and post hoc tests performed by the Bonferroni test.

^a^Significant difference between groups 1 and 2. ^b^Significant difference between groups 1 and 3.

Supplementary Table S2 shows the univariate linear regression of the relationships between the confounding factors and the pulmonary outcomes. Important factors associated with pulmonary function (IVB history, full term, height) and baseline significant factors (factors that differed between groups 2 and 3 included sex, GA, BW, CS, Apgar scores at 1 min and 5 min, BPD, PROM, RDS, received surfactant, duration of mechanical ventilator use after birth, breastfeeding duration, atopic dermatitis, paternal asthma, present paternal smoking) were analyzed. Then, those factors that had significant predictive power for pulmonary function in the univariate model were used for the pretest multivariate regression model. The factors (sex, test age, BPD, RDS, child height and duration of mechanical ventilator use after birth) that had significant predictive power in the pretest multivariable regression model were selected and used to construct the final multivariable model. FEV_1_/FVC was not included in the multivariable regression model because there were no significant factors identified in the univariable regression model.

Table 3 shows the final multivariable regression model of the relationship between confounding factors and pulmonary function. Male sex showed a positive relationship with FVC (*P* < 0.01), FEV_1_ (*P* < 0.01) and FEF_25–75_ (*P* < 0.01). Child age at pulmonary function test showed a negative association with R5 (*P* < 0.01) and R5–R20 (*P* < 0.01). Child height showed a positive association with FVC (*P* < 0.001), FEV_1_ (*P* < 0.001) and FEF_25–75_ (*P* < 0.001). Importantly, a history of IVB treatment showed no significant relationship with any of the spirometry or IOS parameters after adjusting for other confounding factors.

**Table 3:** Multivariate linear regression model of risk factors for pulmonary function parameters.

	FVC	FEV_1_	FEF_25–75_	
Constant (adjusted *R*^*2*^%)	− 2.19 (57.1%)	− 2.26 (59.0%)	− 3.79 (45.1%)	
IVB treatment	0.03 (− 0.10; 0.16)	0.05 (− 0.07; 0.17)	0.14 (− 0.11; 0.38)	
Full term	0.05 (− 0.14; 0.24)	0.05 (− 0.12; 0.22)	0.02 (− 0.33; 0.37)	
Male	0.13 (0.05; 0.20)^a^	0.13 (0.06; 0.20)^a^	0.20 (0.06; 0.35)^a^	
Age at pulmonary function test	4.5E−3 (− 0.10; 0.11)	− 3.3E−3 (− 0.10; 0.09)	− 0.04 (− 0.23; 0.15)	
BPD	− 0.08 (− 0.24; 0.08)	− 0.03 (− 0.18; 0.11)	0.14 (− 0.16; 0.44)	
RDS	− 0.01 (− 0.18; 0.16)	− 0.03 (− 0.18; 0.13)	− 0.23 (− 0.55; 0.09)	
Duration of mechanical ventilation after birth	− 1.0E−3 (− 3.9E−3; 1.8E−3)	− 1.1E−3 (− 3.7E−3; 1.5E−3)	− 2.3E−3 (− 0.01; 3.0E−3)	
Height	0.03 (0.02; 0.04)^b^	0.03 (0.02; 0.04)^b^	0.05 (0.03; 0.06)^b^	

	R5	X5	R20	R5–R20
Constant (adjusted R^2^%)	29.36 (24.6%)	− 11.42 (2.0%)	15.01 (4.3%)	14.35 (23.7%)
IVB treatment	− 0.15 (− 1.64; 1.34)	− 0.14 (− 1.27; 0.99)	− 0.36 (− 1.29; 0.58)	0.21 (− 0.88; 1.29)
Full term	− 0.79 (− 2.94; 1.35)	0.24 (− 1.39; 1.86)	− 0.25 (− 1.60; 1.10)	− 0.54 (− 2.11; 1.02)
Male	− 0.46 (− 1.33; 0.41)	0.44 (− 0.21; 1.10)	− 0.02 (− 0.56; 0.53)	− 0.45 (− 1.08; 0.19)
Age at pulmonary function test	− 1.60 (− 2.77; − 0.43)^a^	0.17 (− 0.72; 1.05)	− 0.19 (− 0.92; 0.55)	− 1.42 (− 2.27; − 0.56)^a^
BPD	0.47 (− 1.35; 2.29)	− 0.46 (− 1.84; 0.91)	0.36 (− 0.78; 1.50)	0.11 (− 1.22; 1.44)
RDS	0.48 (v1.47; 2.42)	0.30 (− 1.17; 1.77)	0.08 (− 1.14; 1.29)	0.40 (− 1.01; 1.82)
Duration of mechanical ventilation after birth	9.6E−4 (− 0.03; 0.03)	0.01 (− 0.01; 0.03)	− 4.4E−3 (− 0.02; 0.02)	0.01 (− 0.02; 0.03)
Height	− 0.07 (− 0.16; 0.03)	0.06 (− 0.01; 0.13)	− 0.06 (− 0.12; 1.1E−3)	− 0.01 (− 0.08; 0.06)

*BPD* bronchopulmonary dysplasia; *FEF*_25–75_, forced expiratory flow between 25 and 75% of FVC; *FEV*_1_ forced expiratory volume in 1 second; *FVC* forced vital capacity; *GA* gestational age; *IVB* intravitreal injection of Bevacizumab; *R5* respiratory resistance at 5 Hz; *R20* respiratory resistance at 20 Hz; *R5-R20* difference between respiratory resistance at 5 Hz and 20 Hz; *X5* respiratory reactance at 5 Hz.

The data are presented as coefficients (95% confience intervals).

^a^P values < 0.01. ^b^P values < 0.001.

## Discussion

Our study revealed that, preterm patients with and without a history of IVB treatment showed similar pulmonary function at the age of 6–7 years. We used 2 lung function assessment tools, namely, spirometry and impulse oscillometry, which measured different components of lung function, to determine the outcome of pulmonary function. Pulmonary function did not reveal a significant difference between these 2 groups of patients, although the IVB group had relatively unfavorable baseline demographics, including reduced GA, reduced BW, reduced Apgar scores, increased percentages of RDS and BPD, and extended periods of ventilator use. Male sex, age and height were the 3 most important factors found to be associated with superior pulmonary function at school age. To the best of our knowledge, this is the first study to examine the pulmonary function of ROP patients at school age after anti-VEGF treatment and compare it with that of patients who had not received such treatment. This information is important for clinicians to consider before administering anti-VEGF to preterm children, who face an increased risk of compromised pulmonary function.

VEGF plays an important role in lung disease and development^12,13,39–41^. In the neonatal lungs, VEGF regulates angiogenesis, interacting closely with vascularization and bronchiolar branching^41^, and enhances type II pneumocyte growth^42^. Animal studies showed that VEGF receptor blockers or a single dose of anti-VEGF impaired pulmonary vascular growth and postnatal alveolarization and caused pulmonary hypertension in infant rats^13,43,44^. Additionally, the complex coordinated growth of lung epithelial cells and vessels requires a normal VEGF gradient, and the disruption of temporal and spatial expression of VEGF disrupts lung morphogenesis^45^. Multiple lung diseases are related to VEGF; for example, asthma, lung cancer, and acute lung injury are related to VEGF overexpression, while emphysema and pulmonary hypertension are related to VEGF receptor blockage^39,40^. Taken together, VEGF is vital for the healthy development of lung structure and function.

Why did the use of anti-VEGF treatment not further compromise lung function in these preterm children compared to patients with no history of anti-VEGF therapy? Although preterm infants with prior anti-VEGF treatment faced an elevated risk of poor lung function, their lung function was similar to that of preterm infants without anti-VEGF treatment by the time they reached school age. First, it is possible that the coordinated timely release of VEGF, rather than a high level of VEGF, was vital to lung vascularity development, and a VEGF blockage alone had no impact on neonatal oxygen requirements^46^. Also, although serum VEGF levels were suppressed for 2 months after the use of IVB^21,22,47^, these suppressive effects are relatively short compared to the full period of lung development. Lung development starts from a postmenstrual age of 4 weeks and continues into early adolescence^48^. More than 85% of alveoli are formed after birth, and the lungs have the potential to continue growing even in adulthood^48,49^. Vollsæter et al. showed a similar slope of lung function development from mid-childhood to adulthood between preterm and term-born groups^50^. Longitudinal studies up to adolescence and 21 years of age also showed gradual improvement in lung function among preterm patients^51,52^. Other studies showed that lung alveolar growth had sufficient plasticity to catch up to normal after impairment by drugs, toxins or malnutrition^53–55^.

Furthermore, in addition to VEGF, there are multiple other factors that regulate lung development, including Wnt signaling, retinoic acid signaling, fibroblast growth factor signaling, and histone acetylation^56^. The impact of altered VEGF levels can be mitigated by complex molecular signaling in lung development. Additionally, VEGF signaling can be regulated by multiple factors, such as platelet-derived growth factor, transforming growth factor, insulin growth factor-I, fibroblast growth factor, keratinocyte growth factor, estrogens and IL-1β^39^. There is also a VEGF homolog known as placental growth factor, whose function is similar to that of VEGF^39^. Future studies need to examine the impact of VEGF suppression on lung homeostasis of various signaling factors.

However, we still cannot fully exclude the possibility that anti-VEGF has a minor effect on the distal airway because VEGF plays an important role in lung alveolarization and type II pneumocyte development^41,42^. FEF_25–75_ is related to dysfunction of the distal airway^57^, and R5–R20 represents distal airway resistance^38^. In our study, we noted that, compared to preterm infants with no history of IVB treatment, preterm infants with a history of IVB anti-VEGF treatment showed a minor trend toward reduced FEF_25–75_ (IVB: 1.12; non-IVB: 1.35; *p* = 0.11) and increased R5–R20 (IVB: 4.12; non-IVB: 4.08; *p* = 1.00), but the difference was not statistically significant. Also, longer mechanical ventilation need was observed in Group 3. However, whether anti-VEGF have temporary detrimental effect to infant in perinatal period was uncertain due to complicated confounding factors such as BPD. Future studies are needed to assess the possibilities of a minor effect of IVB on late lung alveolarization.

This study was limited by being a single-center study with a small sample size and a retrospective study design. Also, only infants who survived to school age were included in our study, which meant that the anti-VEGF effect to those most vulnerable patients with mortality was not accounted in our study. Additionally, patients receiving IVB anti-VEGF treatment alone and in conjunction with laser therapy were combined into the same group due to the small sample size of the subgroup. Ultimately, the *R*^*2*^% value of Table 3 was medium, which meant it was not a perfect model to predict pulmonary function but still provided us with information on the relationship between the variables and pulmonary function. However, our study had the strength of detailed documentation of baseline data and various risk factors. In addition, instead of only one pulmonary function test, two tests including spirometry and impulse oscillometry, were conducted in these children when they reached school age.

In conclusion, preterm infants with a history of IVB treatment showed similar pulmonary outcomes at school age compared to preterm infants without IVB treatment. This is the first study to examine the impact of pulmonary function following anti-VEGF treatment for ROP in preterm children when they reach school age. Our findings do not imply that it is “absolutely safe” to use anti-VEGF in these patients. Judicious use of anti-VEGF for ROP is still recommended until its systemic impact is fully understood. Future prospective randomized studies are needed to confirm the impact of VEGF suppression on the pulmonary function of these vulnerable patients.

## Supplementary Information

Supplementary Information.

## Data Availability

The datasets generated during and/or analyzed during the current study are available from the corresponding authors on reasonable request.
