# Supplementary material for: Pulmonary function in school-age children following intravitreal injection of bevacizumab for retinopathy of prematurity
Source: Sci Rep. 2022 Nov 5;12:18788. doi: 10.1038/s41598-022-22338-2 (PMC9637204; doi:10.1038/s41598-022-22338-2)
Supplement: Supplementary file 1 — Supplementary Information. [file 41598_2022_22338_MOESM1_ESM.docx]

Scientific Reports

Supplementary Information

**Pulmonary function** **in school-age children following intravitreal injection of bevacizumab for retinopathy of prematurity**

Ching-Yen Huang, Shen-Hao Lai, Hsiao-Jung Tseng, Tsung-Chieh Yao, Wei-Chi Wu

**Content**

Supplementary Table S1……………………………………………………2

Supplementary Table S2……………………………………………………4

Supplementary Table S1. Demographics.

|  | **Full-term Control  (Group 1; n= 50)** | **Prematurity without  IVB Treatment (Group 2; n= 33)** | **Prematurity with  IVB Treatment  (Group 3; n= 35)** | ***P-value***  ***(F-value)*** | **Group 2 vs 3** |
| --- | --- | --- | --- | --- | --- |
| **Neonatal period** |  |  |  |  |  |
| Apgar score, mean ± SD |  |  |  |  |  |
| 1 minute | 9.0±0.1^c,d^ | 5.9±2.5^c,e^ | 4.3±1.9^d,e^ | <0.001^a^ (185) | 0.001^e^ |
| 5 minutes | 9.9±0.7^c,d^ | 7.6±2.0^c,e^ | 6.6±1.5^d,e^ | <0.001^a^ (129) | 0.02^e^ |
| **Prenatal period** |  |  |  |  |  |
| PIH, no. (%) | 0 (0.0)^c^ | 3 (9.1)^c^ | 2 (5.7) | 0.04^b^ | 0.56 |
| GDM, no. (%) | 0 (0.0) | 1 (3.0) | 1 (2.9) | 0.28^b^ | 0.94 |
| Preeclampsia, no. (%) | 0 (0.0)^c^ | 4 (12.1)^c^ | 1 (2.9) | 0.006^b^ | 0.13 |
| APH, no. (%) | 0 (0.0)^c,d^ | 11 (33.3)^c^ | 15 (42.9)^d^ | <0.001^b^ | 0.48 |
| Placental abruption, no. (%) | 0 (0.0) | 2 (6.1) | 1 (2.9) | 0.09^b^ | 0.49 |
| Vitamin D supplement during pregnancy, no. (%) |  |  |  | 0.33^b^ | 0.76 |
| Never | 43 (86.0) | 23 (69.7) | 26 (76.5) |  |  |
| <3 months | 3 (6.0) | 2 (6.1) | 2 (5.9) |  |  |
| ≥3 months | 4 (8.0) | 8 (24.2) | 6 (17.6) |  |  |
| **Postnatal period** |  |  |  |  |  |
| MAS, no. (%) | 0 (0.0) | 0 (0.0) | 0 (0.0) |  |  |
| RDS, no. (%) | 0 (0.0)^c,d^ | 20 (60.6)^c,e^ | 30 (85.7)^d,e^ | <0.001^b^ | 0.007^e^ |
| PVL, no. (%) | 0 (0.0) | 0 (0.0) | 0 (0.0) |  |  |
| NEC, no. (%) | 0 (0.0)^c^ | 3 (9.1)^c^ | 2 (5.7) | 0.04^b^ | 0.56 |
| Breastfeeding, no. (%) |  |  |  | 0.06^b,d,e^ | 0.03^e^ |
| Never | 13 (26.0) | 7 (21.2) | 0 (0.0) |  |  |
| < 3 months | 12 (24.0) | 6 (18.2) | 8 (22.9) |  |  |
| 3~6 months | 10 (20.0) | 9 (27.3) | 9 (25.7) |  |  |
| > 6 months | 15 (30.0) | 11 (33.3) | 18 (51.4) |  |  |
| **Allergic diseases, no. (%)** |  |  |  |  |  |
| Atopic dermatitis | 11 (22.4)^c^ | 15 (46.9)^c,e^ | 5 (14.3)^e^ | 0.007^b^ | 0.003^e^ |
| Allergic rhinitis | 28 (56.0) | 20 (60.6) | 20 (57.1) | 0.92^b^ | 0.67 |
| **Smoking exposure, no. (%)** |  |  |  |  |  |
| Paternal smoking during pregnancy | 14 (28.0)^d^ | 9 (27.3)^e^ | 20 (57.1)^d,e^ | 0.007^b^ | 0.007^e^ |
| Family member smoking during pregnancy | 18 (36.0)^d^ | 17 (51.5) | 20 (57.1)^d^ | 0.13^b^ | 0.57 |
| Current paternal smoking | 13 (26.0) | 10 (30.3) | 14 (40.0) | 0.39^b^ | 0.33 |
| Current maternal smoking | 0 (0.0)^d^ | 1 (3.0) | 2 (5.7)^d^ | 0.26^b^ | 0.35 |
| Current family member smoking | 13 (26.0)^d^ | 15 (45.5) | 17 (48.6)^d^ | 0.06^b^ | 0.71 |
| **Paternal education, no. (%)** |  |  |  | 0.32^b^ | 0.31 |
| Elementary school | 0 (0.0) | 0 (0.0) | 1 (2.9) |  |  |
| Junior high school | 0 (0.0) | 0 (0.0) | 3 (8.6) |  |  |
| Senior high school | 10 (20.4) | 6 (18.2) | 4 (11.4) |  |  |
| College | 27 (55.1) | 19 (57.6) | 21 (60.0) |  |  |
| Graduate degree | 12 (24.5) | 8 (24.2) | 6 (17.1) |  |  |
| **Maternal education, no. (%)** |  |  |  | 0.23^b^ | 0.27 |
| Elementary school | 0 (0.0) | 0 (0.0) | 0 (0.0) |  |  |
| Junior high school | 1 (2.0) | 1 (3.0) | 2 (5.7) |  |  |
| Senior high school | 3 (6.0) | 6 (18.2) | 4 (11.4) |  |  |
| College | 41 (82.0) | 25 (75.8) | 23 (65.7) |  |  |
| Graduate degree | 5 (10.0) | 1 (3.0) | 6 (17.1) |  |  |
| **Household income (USD), no. (%)** |  |  |  | 0.35^b^ | 0.26 |
| <10,000 | 3 (6.0) | 1 (3.0) | 1 (2.9) |  |  |
| 10,000-20,000 | 11 (22.0) | 8 (24.2) | 6 (17.6) |  |  |
| 20,000-30,000 | 7 (14.0) | 5 (15.2) | 10 (29.4) |  |  |
| 30,000-40,000 | 8 (16.0) | 11 (33.3) | 5 (14.7) |  |  |
| >40,000 | 21 (42.0) | 8 (24.2) | 12 (35.3) |  |  |
| **Maternal nutritional status** |  |  |  |  |  |
| Maternal height (cm), mean ± SD | 160.8±4.8^c^ | 157.2±4.9^c^ | 159.5±5.2 | 0.006^a^ (8) | 0.16 |
| Maternal weight (kg), mean ± SD | 57.9±8.5 | 55.5±8.2 | 58.1±11.6 | 0.39^a^ (2) | 0.78 |
| Maternal BMI, mean ± SD | 22.4±3.2 | 22.5±2.8 | 22.8±4.1 | 0.79^a^ (0.2) | 1.00 |
| **ROP status** |  |  |  |  |  |
| ROP stage, eyes, no. (%) |  |  |  | <0.001^f^ |  |
| stage 1 | 0 (0.0) | 4 (14.3) | 0 (0.0) |  |  |
| stage 2 | 0 (0.0) | 16 (57.1) | 6 (8.7) |  |  |
| stage 3 | 0 (0.0) | 8 (28.6) | 63 (91.3) |  |  |
| ROP zone, eyes, no. (%) |  |  |  | <0.001^f^ |  |
| zone 1 | 0 (0.0) | 4 (14.3) | 10 (14.5) |  |  |
| zone 2 | 0 (0.0) | 20 (71.4) | 59 (85.5) |  |  |
| zone 3 | 0 (0.0) | 4 (14.3) | 0 (0.0) |  |  |
| Plus disease, eyes, no. (%) | 0 (0.0) | 14 (50.0) | 61 (88.4) | <0.001^f^ |  |
| ROP treatment, eyes, no. (%) |  |  |  | <0.001^f^ |  |
| No ROP | 100 (100.0) | 38 (57.6) | 0 (0.0) |  |  |
| ROP without treatment | 0 (0.0) | 14 (21.2) | 0 (0.0) |  |  |
| Laser | 0 (0.0) | 14 (21.2) | 0 (0.0) |  |  |
| IVB | 0 (0.0) | 0 (0.0) | 44 (63.8) |  |  |
| IVB + Laser | 0 (0.0) | 0 (0.0) | 25 (36.2) |  |  |

Abbreviations: *APH*, antepartum hemorrhage; *BMI*, body mass index; *GDM*, gestational diabetes mellitus; *IVB*, intravitreal injection of Bevacizumab; *MAS*, meconium aspiration syndrome; *NEC*, necrotizing enterocolitis; *PHTN*, pulmonary hypertension; *PIH*, pregnancy induced hypertension; *PVL*, periventricular leukomalacia; *RDS*, respiratory distress syndrome; *SD*, standard deviation.

^a^P values calculated by analysis of variance and post hoc tests performed by the Bonferroni test.

^b^P values calculated by chi-square test or Fisher exact test.

^c^Significant difference between groups 1 and 2.

^d^Significant difference between groups 1 and 3.

^e^Significant difference between groups 2 and 3.

^f^Group 1 was excluded.

Supplementary Table S2. Univariate linear regression model of risk factors for pulmonary function parameters.

|  | **FVC** | **FEV_1_** | **FEV_1_/FVC** | **FEF_25-75_** |
| --- | --- | --- | --- | --- |
| IVB group^d^ | 0.08 (-0.05; 0.21) | 0.09 (-0.03; 0.21) | 1.81 (-1.58; 5.20) | 0.23 (0.02; 0.45)^a^ |
| Full term group^d^ | 0.28 (0.16; 0.39)^c^ | 0.26 (0.16; 0.37)^c^ | 0.84 (-2.24; 3.92) | 0.45 (0.25; 0.64)^c^ |
| Male | 0.09 (-0.02; 0.19) | 0.09 (-0.01; 0.18) | 0.89 (-1.69; 3.48) | 0.13 (-0.05; 0.30) |
| Gestational age | 0.02 (0.01; 0.03)^c^ | 0.02 (0.01; 0.03)^c^ | 0.02 (-0.20; 0.23) | 0.03 (0.02; 0.04)^c^ |
| Birth weight | 1.2E-4 (1.4E-8; 8.6E-5)^c^ | 1.2E-4 (8.2E-5; 1.6E-4)^c^ | 2.5E-4 (-9.1E-4; 1.4E-3) | 1.9E-4 (1.2E-4; 2.6E-4)^c^ |
| Cesarean section | 0.01 (-0.10; 0.12) | 0.01 (-0.09; 0.12) | 0.36 (-2.32; 3.04) | 0.00 (-0.18; 0.18) |
| Apgar score at 1 minute | 0.06 (0.04; 0.08)^c^ | 0.05 (0.04; 0.07)^c^ | -0.07 (-0.58; 0.44) | 0.08 (0.05; 0.11)^c^ |
| Apgar score at 5 minutes | 0.07 (0.04; 0.09)^c^ | 0.06 (0.04; 0.08)^c^ | -0.03 (-0.71; 0.64) | 0.10 (0.05; 0.14)^c^ |
| PROM | -0.05 (-0.18; 0.09) | -0.04 (-0.16; 0.08) | 0.45 (-2.76; 3.67) | -0.06 (-0.28; 0.16) |
| BPD | -0.32 (-0.42; -0.22)^c^ | -0.29 (-0.38; -0.19)^c^ | 1.00 (-1.87; 3.86) | -0.40 (-0.59; -0.22)^c^ |
| RDS | -0.25 (-0.35; -0.15)^c^ | -0.23 (-0.33; -0.14)^c^ | -0.37 (-3.08; 2.34) | -0.40 (-0.57; -0.23)^c^ |
| Received surfactant | -0.28 (-0.38; -0.17)^c^ | -0.27 (-0.36; -0.17)^c^ | -1.20 (-3.97; 1.56) | -0.45 (-0.62; -0.28)^c^ |
| Duration of mechanical ventilation after birth | -4.0E-3 (-0.01; -2.6E-3)^c^ | -3.7E-3 (-4.9E-3; -2.5E-3)^c^ | -0.01 (-0.04; 0.03) | -0.01 (-0.01; -3.7E-3)^c^ |
| Breastfeeding duration | -0.03 (-0.07; 0.02) | -0.03 (-0.08; 0.01) | -0.93 (-2.08; 0.21) | -0.07 (-0.15; 0.01) |
| Height | 0.03 (0.02; 0.04)^c^ | 0.03 (0.02; 0.04)^c^ | 0.13 (-0.08; 0.34) | 0.04 (0.03; 0.06)^c^ |
| Atopic dermatitis | 0.01 (-0.11; 0.13) | -0.02 (-0.13; 0.09) | -2.24 (-5.16; 0.68) | -0.07 (-0.27; 0.13) |
| Paternal asthma | 0.03 (-0.17; 0.23) | 0.06 (-0.13; 0.25) | 2.70 (-2.36; 7.77) | 0.31 (-0.03; 0.66) |
| Current paternal smoking | -0.13 (-0.24; -0.02)^a^ | -0.12 (-0.22; -0.02)^a^ | -0.47 (-3.16; 2.22) | -0.14 (-0.32; 0.04) |
|  | **R5** | **X5** | **R20** | **R5-R20** |
| IVB group^d^ | -0.11 (-1.28; 1.05) | -0.48 (-1.28; 0.33) | -0.08 (-0.82; 0.66) | -0.03 (-0.84; 0.79) |
| Full term group^d^ | -1.56 (-2.62; -0.51)^b^ | -0.24 (-0.98; 0.49) | -0.42 (-1.09; 0.26) | -1.15 (-1.89; -0.40)^b^ |
| Male | -0.27 (-1.19; 0.66) | 0.48 (-0.13; 1.09) | -0.03 (-0.60; 0.53) | -0.24 (-0.89; 0.42) |
| Gestational age | -0.13 (-0.20; -0.06)^c^ | -8.5E-4 (-0.05; 0.05) | -0.03 (-0.08; 0.01) | -0.10 (-0.15; -0.05)^c^ |
| Birth weight | -8.2E-4 (-1.2E-3; -4.3E-4)^c^ | 7.6E-5 (-2.0E-4; 3.5E-4) | -2.7E-4 (-5.2E-4; -1.6E-5)^a^ | -5.5E-4 (-8.3E-4; -2.8E-4)^c^ |
| Cesarean section | 0.96 (0.03; 1.89)^a^ | -0.53 (-1.15; 0.09) | 0.47 (-0.05; 1.00) | 0.49 (-0.19; 1.16) |
| Apgar score at 1 minute | -0.31 (-0.49; -0.14)^c^ | 0.04 (-0.08; 0.16) | -0.08 (-0.18; 0.02) | -0.24 (-0.36; -0.12)^c^ |
| Apgar score at 5 minutes | -0.38 (-0.61; -0.16)^c^ | 0.03 (-0.13; 0.19) | -0.11 (-0.25; 0.02) | -0.27 (-0.43; -0.11)^c^ |
| PROM | 0.77 (-0.37; 1.91) | -0.16 (-0.92; 0.60) | 0.04 (-0.60; 0.68) | 0.73 (-0.09; 1.55) |
| BPD | 1.93 (0.98; 2.89)^c^ | -0.07 (-0.75; 0.61) | 0.58 (0.02; 1.14)^a^ | 1.35 (0.65; 2.05)^c^ |
| RDS | 1.61 (0.70; 2.53)^b^ | 0.17 (-0.48; 0.81) | 0.35 (-0.18; 0.89) | 1.26 (0.60; 1.92)^c^ |
| Received surfactant | 1.89 (0.97; 2.81)^c^ | 0.04 (-0.61; 0.70) | 0.56 (0.02; 1.10)^a^ | 1.33 (0.66; 2.00)^c^ |
| Duration of mechanical ventilation after birth | 0.02 (0.01; 0.03)^b^ | 2.7E-3 (-0.01; 0.01) | 0.01 (-1.6E-3; 0.01) | 0.02 (0.01; 0.02)^b^ |
| Breastfeeding duration | 0.36 (-0.05; 0.76) | 0.08 (-0.19; 0.36) | 0.17 (-0.08; 0.43) | 0.18 (-0.11; 0.47) |
| Height | -0.18 (-0.25; -0.11)^c^ | 0.06 (0.02; 0.11)^a^ | -0.08 (-0.12; -0.03)^b^ | -0.11 (-0.16; -0.06)^c^ |
| Atopic dermatitis | 0.51 (-0.53; 1.56) | -0.77 (-1.45; -0.08)^a^ | 0.33 (-0.31; 0.97) | 0.18 (-0.56; 0.93) |
| Paternal asthma | -0.33 (-2.17; 1.50) | -0.47 (-1.69; 0.74) | 0.73 (-0.37; 1.84) | -1.07 (-2.35; 0.21) |
| Current paternal smoking | 0.11 (-0.85; 1.08) | 0.09 (-0.55; 0.72) | 0.36 (-0.22; 0.95) | -0.25 (-0.93; 0.43) |

Abbreviations: *BPD*, bronchopulmonary dysplasia; *FEF_25-75_*, forced expiratory flow between 25% and 75% of FVC; *FEV_1_*, forced expiratory volume in first second; *FEV_1_/FVC*, ratio of FEV_1_ to FVC; *FVC*, forced vital capacity; *GA*, gestational age; *IVB*, intravitreal injection of Bevacizumab; *PROM*, premature rupture of membrane; *R5*, respiratory resistance at 5 Hz; *R20*, respiratory resistance at 20 Hz; *R5-R20*, difference between respiratory resistance at 5 Hz and 20 Hz; *RDS*, respiratory distress syndrome; *X5*, respiratory reactance at 5 Hz.

The data are presented as coefficients (95% confidence intervals).

^a^P values <0.05.

^b^P values <0.01.

^c^P values <0.001.

^d^Difference between R5 and R20.

^e^Reference group is the prematurity without IVB group.
